# Supplementary material for: Cross-Cultural Adaptation and Validation of the Perceptions of Empowerment in Midwifery Scale in the Spanish Context (PEMS-e)
Source: Healthcare (Basel). 2023 May 18;11(10):1464. doi: 10.3390/healthcare11101464 (PMC10218177; doi:10.3390/healthcare11101464)
Supplement: Supplementary file 1 [file healthcare-11-01464-s001.zip › healthcare-2350891-supplementary/Table S1 Studies using the PEMS and PEMS-R scales.pdf]

Table S1. Studies using the PEMS and PEMS-R scales

Study/Year/ Country	Design/ Aim	Sample/ Adequacy of the simple to factorial analysis	Items removed	Scale Obtained/Used	Scale operation of PEMS/PEMS-R	Psychometric Properties	Findings/Comments
Mathews et al. 2009.[20] **Ireland**	-Study of the development and validation of a scale<br>-Design based on the *Understanding of Empowerment Scale (UES)* generating 22 items<br>-Pilot study with 20 midwives<br>-Construct validity by means of AFE (Principal axis factoring and Direct Oblimin rotation method).<br>-Convergent validity with the *Conditions for Work Effectiveness Questionnaire (CWEQ*),<br>-Discriminant validity between sub-groups (grade-role, years of experience)	N=244<br>KMO:0.838	Items 11, 14, 15 and 19 were removed for loadings below 0.4	***Perceptions of Empowerment in Midwifery Scale (PEMS*)**<br>18 items organised in 3 subescales:<br>-Autonomous practice (6 items),<br>-Effective management (6 items),<br>-Women-centred practice (6 items)	Likert scale where each item is scored from 1 to 5.<br>Some items are reverse-scored.<br>For each subescale, the score for each item is added up and divided by the number of items in the subescale, so that each subescale can have a score from 1 to 5.<br>The higher score on the scale, the lower empowerment.	Autonomous practice: α Cronbach 0.77<br>Effective management: α Cronbach 0.82<br>Women-centred practice: α Cronbach 0.66<br>A moderate correlation between the PEMS and the CWEQ was observed (r=0.589), which supports construct validity.	
Henriques et al. 2012.[23] **Portugal**	-Validation study of a scale. Transcultural adaptation of the PEMS into Portuguese (4 bilingual translators).<br>-Evaluation by 6 experts<br>-Logical Validity with 4 *Enfermeiros Especialistas em Enfermagem de Saúde Materna e Obstétri*ca.<br>-Correlation study by calculating standardised reliability coefficients in relation to the original PEMS.<br>-Construct validity by means of EFA (Principal Component Extraction Method and Varimax rotation).	N=309<br>KMO:0.719	Items 3, 4, 7, 17 and 18 were removed as they obtained a low correlation in the standardised reliability coefficients with the original scale (below 0.200)	***Escala da Perceção do Empoderamento dos Enfermeiros Especialistas em Enfermagem de Saúde Materna e Obstétrica' – PEMS-PT.***<br>17 items organised in 5 subescales:<br>-Effective management and Interdisciplinary relations (9 items),<br>-Sustained and autonomous practice(4 items),<br>-Professional communication and consent (2 items),<br>-Recognition team	Likert scale where each item is scored from 1 to 5.<br>Total score between 17 to 85 points.<br>The higher score on the scale, the lower empowerment<br>The average value is calculated by adding the maximum value + the minimum value /2 + the minimum value.<br>Values identical to the mean value will be considered as medium level of empowerment, above this value above is considered as low level of empowerment and below is considered as high level of empowerment.	α Standardised Cronbach's 0.832	

				health (1 items),<br>-Education and training (1 items))		
***Pallant et al. 2015.*[16] Nueva Zeland**	-Validation study of a scale.<br>-Construct validity using EFA (Principal Component Extraction Method and Oblimin rotation, parallel analysis). The final solution was tested by Principal Axes Factorisation.<br>-Analysis by known groups (idea of leaving the profession in the last six months versus no idea).	N=600<br>KMO:0.88	Items 13 and 15 were removed for loadings below 0.4 and item 10 was removed for loadings on more than one factor.	***Perceptions of Empowerment in Midwifery Scale - Revised (PEMS-R)***<br>19 items organised in 4 subescales:<br>- Authonomy/ empowerment (4 items)<br>-Manager support (5 items),<br>-Professional recognition (5 items)<br>-Skills and resources (5 items)	Likert scale where each item is scored from 1 to 5.<br>Some items are reverse-scored.<br>For each subescale, the score for each item is added up and divided by the number of items in the subescale, so that each subescale can have a score from 1 to 5.<br>The higher score on the scale, the higher empowerment.	Authonomy/ empowerment α Cronbach 0.75 Mean inter-item correlation 0.46<br>Manager support α Cronbach 0.88 Mean inter-item correlation 0.59<br>Professional recognition α Cronbach 0.76 Mean inter-item correlation 0.38<br>Skills and resources α Cronbach 0.55 Mean inter-item correlation 0.20<br>Statistically significant differences for the 4 subescales in the analysis known groups by t student
***Lukasse and Pajalic 2016.*[21] Norwey**	-Validation study of a scale<br>-Construct validity using EFA (Principal Component Extraction , axis factoring and Direct Oblimin rotation)<br>-Inferential analysis between various variables studied (age, academic degree, years of experience, shifts and hours of work, number of deliveries, area of work).	N=595<br>KMO:0.877	Items 7 and 21 were removed for loadings below 0.4	***Perceptions of Empowerment in Midwifery Scale (PEMS)***<br>20 items organised in 3 subescales:<br>-Supportive management (7 items)<br>-Autonomous profesional role (9	Likert scale where each item is scored from 1 to 5.<br>Some items are reverse-scored.<br>For each subescale, the score for each item is added up and divided by the number of items in the subescale, so that each subescale can have a score from 1 to 5.<br>The higher score on the scale, the higher empowerment	- Supportive management α Cronbach 0.868 Mean inter-item correlation 0.48<br>- Autonomous profesional role α Cronbach 0.761 Mean inter-item correlation 0.26<br>- Equipped for practice α Cronbach 0.619 Mean inter-item correlation 0.29

				items)<br>-Equipped for practice (4 items)			
***Hildingsson et al. 2016.*[10]**<br>**Australia, New Zealand and Sweden.**	-Cross-sectional survey<br>-Aim: To compare the perception of midwives' empowerment in 3 countries (Australia, New Zealand and Sweden).<br>*-Perceptions of Empowerment in Midwifery Scale -Revised (PEMS-R)* was used	N=2585<br>n=1037 (Australia)<br>n=1073<br>(New Zealand)<br>n=475 (Sweden)	N/A	***Perceptions of Empowerment in Midwifery Scale - Revised (PEMS-R)***<br>19 items organised in 4 subescales:<br>- Authonomy/ empowerment (4 items)<br>-Manager support (5 items),<br>-Professional recognition (5 items)<br>-Skills and resources (5 items)	Likert scale where each item is scored from 1 to 5.<br>Some items are reverse-scored.<br>For each subescale, the score for each item is added up and divided by the number of items in the subescale, so that each subescale can have a score from 1 to 5.<br>The higher score on the scale, the higher empowerment.	N/A	Higher perception of empowerment among Swedish midwives<br>In the subscales de Authonomy/ empowerment, Manager support and Professional recognition with M(SD) 4.34(0.48)/ 3.69(0.79)/ 4.27(0.74) respectively, but less empowerment in Skills and resources with M(SD)=3.78(0.59)
***Murat Öztürk et al. 2018.*[24]**<br>**Turkey**.	-Validation study of a scale.<br>-Transcultural adaptation of the PEMS into turkish (3 bilingual translators)<br>-Evaluation by 3 experts.<br>-Construct validity using EFA (no details of the method used are given)	N=135<br>KMO:0.783	Items 3,12 and 22 were removed for loadings below 0.30	***Perceptions of Empowerment in Midwifery Scale (PEMS) Turkish version***<br>19 items organised in 3 subescales:<br>-Support and management (6 items)<br>-Skill (6 items)<br>-Resource (7 items)	Likert scale where each item is scored from 1 to 5.<br>Total score from 19 to 95 points, but the sense of the score is not specified.	Global α Cronbach 0.823 (before removing items 3, 12 and 22).<br>- Support and management α Cronbach 0.706<br>-Skill α Cronbach 0.794<br>-Resource α Cronbach 0.718	The initial EFA proposed a 6-factor solution with poor fit. They performed a new EFA with the original model of *Mathews et al. 2009*.

Fenwick et al. 2018.[22]<br>**Australia**	-Cross-sectional survey<br>-Aim: To assess the professional and emotional well-being of Australian midwives by comparing those who provide continuity of care versus those who do not.	N=862	N/A	***Perceptions of Empowerment in Midwifery Scale - Revised (PEMS-R)***<br>19 items organised in 4 subescales:<br>- Authonomy/ empowerment (4 items)<br>-Manager support (5 items),<br>-Professional recognition (5 items)<br>-Skills and resources (5 items).<br>***Copenhaguen Burnout Inventory (CBI)* and *Depression, Anxiety*** *and* ***Stress Scale-21* (DASS-21)** was used	Likert scale where each item is scored from 1 to 5.<br>Some items are reverse-scored.<br>For each subescale, the score for each item is added up and divided by the number of items in the subescale, so that each subescale can have a score from 1 to 5.<br>The higher score on the scale, the higher empowerment.	N/A	Main results with the PEMS-R<br>The two groups showed significantly different scores on two of the four subscales<br>The continuity group recorded significantly higher levels of empowerment particularly on the Authonomy/ empowerment subscale ($p<0.001$) with a medium effect size ($r= 0.45$), and the Skills and resources subscale ($p=0.002$) which produced a small effect ($r=0.11$).
Sheehy et al. 2019.[11]<br>**Australia**	-Survey (mainly quantitative data with limited text-based opportunity to respond) and Follow-up study in 2013-2014.<br>-Participants recruited during their midwifery training (2007-2008).<br>-Aim: To assess midwives' experiences 6-7 years after completing their studies, differentiating between whether their training had been as a postgraduate nursing degree or as a university degree.<br>-Also to assess burnout, experience and level of empowerment.	N=75	N/A	***Perceptions of Empowerment in Midwifery Scale (PEMS) original***<br>18 items organised in 3 subescales:<br>-Autonomous practice (6 items),<br>-Effective management (6 items),<br>-Women-centred practice (6 items)<br>***Maslach Burnout Inventory (MBI)*** and ***Practice Environment Scale-Nursing Work Index (PES-NWI)***	Likert scale where each item is scored from 1 to 5.<br>Some items are reverse-scored.<br>For each subescale, the score for each item is added up and divided by the number of items in the subescale, so that each subescale can have a score from 1 to 5.<br>The higher score on the scale, the lower empowerment.	N/A	No significant differences were observed between the cohorts.<br>In general, all participants were at a high level of empowerment.<br>There were only differences between professionals who provided continuity versus those who did not, with the former being more empowered on the Autonomous practice and Women-centred practice subscales.

Hajiesmaello et al. 2020.*[25] *Persia	-Validation study of a scale<br>-Transcultural adaptation of the PEMS into persian (4 bilingual translators)<br>-Logical validity (face validity): level of difficulty, relevance and ambiguity.<br>-Content validity using 10 experts (midwifery teachers) and calculation of the CVR according to Lawshe's method and CVI<br>-Construct validity using EFA and CFA (Maximum-Likelihood Estimation method with varimax rotation and parallel analysis according to Horn's system).	N=380<br>KMO: 0.797	Items 2, 7, 9, 11 and 18 were removed without specifying the retention criterion.	***Perceptions of Empowerment in Midwifery Scale (PEMS Persian version***<br>17 items organised in 5 subescales:<br>-Effective management (4 items)<br>-Professional practice (4 items),<br>-Authority (3 items)<br>-Advocacy (3 items)<br>-Professional informant (3 items)	Likert scale where each item is scored from 1 to 5.<br>For each dimension, the score for each item is added up and divided by the number of items in the dimension, so that each dimension can have a score of 1 to 5.<br>For calculate the total score, the scores for each dimension are added together, ranging from 5 to 25. The higher score on the scale, the higher empowerment.	CVR=0.91 for the full scale<br>CVI=0.93 for the full scale, ranging from 0.83 to 1 for each item<br>- Effective management α Cronbach 0.870 coefficient Ω 0.873<br>- Professional practice α Cronbach 0.709 coeficient Ω 0.719<br>- Authority α Cronbach 0.719 coefficient Ω 0.728<br>- Advocacy α Cronbach 0.736 coefficient Ω 0.740<br>- Professional informant α Cronbach 0.736 coefficient Ω 0.745<br>RMSEA 0.039 AGFI 0.931<br>GFI 0.951 CFI 0.958	
Fumagalli et al. 2022.*[26] *Italy	-Validation study of a scale<br>-Transcultural adaptation of the PEMS-R into italian (3 bilingual translators)<br>-Evaluation with a group of 5 expert midwives.<br>-EFA (no data is given on the method used).<br>-Analysis by known groups (midwives' education background and/or years of experience).	N=147	None	***Perceptions of Empowerment in Midwifery Scale - Revised-versión italian (PEMS-R-IT)***<br>- Authonomy/ empowerment (4 items)<br>-Manager support (5 items),<br>-Professional recognition (5 items)<br>-Skills and resources (5 items)	Likert scale where each item is scored from 1 to 5.<br>Some items are reverse-scored.<br>For each subescale, the score for each item is added up and divided by the number of items in the subescale, so that each subescale can have a score from 1 to 5.<br>The higher score on the scale, the higher empowerment.	Autonomy/empowerment α Cronbach 0.807<br>Manager support α Cronbach 0.948<br>Professional recognition α Cronbach 0.866<br>Skills and resources α Cronbach 0.767<br>No statistically significant differences were observed for the 4 subescales in the analysis by known groups with t student.	They report that their EFA shows the same result as that of *Pallant et al. (2015)* identifying the same 4 subscales<br>They do not provide KMO, factor extraction system, model fit values or Cronbach's α coefficient value for the total scale.

AGFI= Adjusted Goodness of Fit Index
CFA=Confirmatory factorial Analysis
CFI= Comparative Fit Index
CVI= Content Validity Index
CVR= Content Validity Ratio
EFA=Exploratory Factorial Analysis
GFI= Goodness of Fit Index
KMO=Kaiser Meyer Olkin
M= Media
N/A: Not applicable
SD= Standard deviation
RMSEA= Root Mean Square Error of Approximation
Ω=Omega Coefficient
α=Cronbach Coefficient
