# Supplementary material for: Cross-Cultural Adaptation and Validation of the Perceptions of Empowerment in Midwifery Scale in the Spanish Context (PEMS-e)
Source: Healthcare (Basel). 2023 May 18;11(10):1464. doi: 10.3390/healthcare11101464 (PMC10218177; doi:10.3390/healthcare11101464)
Supplement: Supplementary file 1 [file healthcare-11-01464-s001.zip › healthcare-2350891-supplementary/Table S2 Scores of Face validity.pdf]

Table S2: Scores of Face validity

Nº Item	Midwife 1	Midwife 2	Midwife 3	Midwife 4	Midwife 5	Midwife 6	Midwife 7	Midwife 8	Midwife 9	Midwife 10	Total*
Item 1	4	4	2	2	4	4	4	3	1	4	3.2
Item 2	4	4	3	3	4	4	4	4	4	4	3.8
Item 3	4	4	4	2	4	2	4	4	4	4	3.6
Item 4	4	4	2	3	4	4	4	4	4	4	3.7
Item 5	4	4	2	2	4	4	4	3	2	4	3.3
Item 6	4	4	3	3	4	3	4	2	4	4	3.5
Item 7	4	4	3	2	4	2	4	2	4	4	3.3
Item 8	4	4	3	2	4	4	4	3	4	4	3.6
Item 9	4	4	2	2	4	4	4	4	2	4	3.4
Item 10	4	4	2	3	4	4	4	4	2	4	3.5
Item 11	4	4	4	3	4	4	4	4	4	4	3.9
Item 12	4	4	3	2	4	4	4	4	4	4	3.7
Item 13	4	4	4	2	4	4	4	4	2	4	3.6
Item 14	2	4	2	1	4	4	4	4	4	4	3.3
Item 15	4	4	4	3	4	4	4	4	4	4	3.9
Item 16	4	4	3	2	4	3	4	3	4	4	3.4
Item 17	4	4	4	3	4	4	3	4	3	4	3.7
Item 18	4	4	3	3	4	4	4	4	4	4	3.8
Item 19	4	4	2	3	4	4	4	3	4	4	3.6
Item 20	4	4	4	3	4	4	4	2	4	4	3.7
Item 21	4	4	4	3	4	4	4	4	2	4	3.7
Item 22	4	4	3	3	4	4	4	3	3	4	3.6

**The total score was calculated by adding the value given to each item by all participating midwives and dividing by the number of midwives (10). A mean of 2 was considered sufficient to consider the item relevant
