# Supplementary material for: Cross-Cultural Adaptation and Validation of the Perceptions of Empowerment in Midwifery Scale in the Spanish Context (PEMS-e)
Source: Healthcare (Basel). 2023 May 18;11(10):1464. doi: 10.3390/healthcare11101464 (PMC10218177; doi:10.3390/healthcare11101464)
Supplement: Supplementary file 1 [file healthcare-11-01464-s001.zip › healthcare-2350891-supplementary/Table S4 Measure of Sampling Adequacy of the EFA with 17 items.pdf]

Table S4 Measure of Sampling Adequacy of the EFA with 22 items.

Items	*Relative Difficulty Index (RDI)*	*Measure of Sampling Adequacy (MSA)*	**Bootstrap 95% Confidence Interval (IC95%)**
**Item 10**	0.51829	0.93450	(0.866 - 0.955)
**Item 8**	0.55427	0.93425	(0.845 - 0.942)
**Item 19**	0.55854	0.93845	(0.775 - 0.955)
**Item 6**	0.58780	0.96360	(0.907 - 0.966)
**Item 21**	0.58902	0.94748	(0.888 - 0.956)
**Item 5**	0.59512	0.90958	(0.841 - 0.926)
**Item 9**	0.59634	0.94019	(0.864 - 0.952)
**Item 22**	0.60000	0.87747	(0.789 - 0.900)
**Item 1**	0.60671	0.92941	(0.861 - 0.941)
**Item 16**	0.61585	0.85525	(0.762 - 0.882)
**Item 7**	0.62622	0.92363	(0.781 - 0.945)
**Item 13**	0.63780	0.92994	(0.814 - 0.950)
**Item 20**	0.64878	0.91343	(0.823 - 0.947)
**Item 17**	0.73720	0.87320	(0.749 - 0.902)
**Item 14**	0.78171	0.91762	(0.765 - 0.934)
**Item 12**	0.82866	0.88187	(0.600 - 0.899)
**Item 15**	0.92866	0.79022	(0.571 - 0.833)

***Number of items proposed to be removed based on MSA: None

Measure of Sampling Adequacy (MSA): Values of MSA below 0.50 suggest that the item does not measure the same domain as the remaining items in the pool, and so that it should be removed.

Relative Difficulty Index (RDI): it assesses the position of the items. For a normal-range test, an optimal pool of items should have about 75% RDI values between .40 and .60 and the remaining values evenly distributed in both tails.
