# Supplementary material for: Cross-Cultural Adaptation and Validation of the Perceptions of Empowerment in Midwifery Scale in the Spanish Context (PEMS-e)
Source: Healthcare (Basel). 2023 May 18;11(10):1464. doi: 10.3390/healthcare11101464 (PMC10218177; doi:10.3390/healthcare11101464)
Supplement: Supplementary file 1 [file healthcare-11-01464-s001.zip › healthcare-2350891-supplementary/Table S5 Final Spanish Version of Perceptions of Empowerment in Midwifery Scale (PEMS-e).pdf]

Número Ítem	Pregunta-Ítem	Totalmente de acuerdo	De acuerdo	Ni de acuerdo ni en desacuerdo	En desacuerdo	Totalmente en desacuerdo
1	**Soy valorada por mi supervisora/dirección**					
5	**Tengo el respaldo de mi supervisora/dirección**					
6	**Mi supervisora/dirección no reconoce mi contribución al cuidado de las mujeres**[R]					
7	**Tengo acceso adecuado a los recursos para las mujeres que están bajo mi cuidado**					
8	**No tengo una supervisora/dirección que me apoye**[R]					
9	**Tengo una comunicación efectiva con la supervisión/dirección**					
10	**No estoy informada sobre los cambios en mi organización que afectarán a mi práctica profesional**[R]					
12	**Tengo el apoyo de mis compañeros de trabajo**					
13	**Tengo la capacidad de decir que no cuando lo considero necesario**					
14	**No se cual es el alcance de mi práctica profesional**[R]					
15	**Soy responsable de mi práctica profesional**					
16	**Soy reconocida como profesional por la profesión médica**					
17	**Tengo control sobre mi práctica profesional**					
19	**No tengo acceso adecuado a los recursos para la formación continuada**[R]					
20	**Tengo autonomía en mi práctica profesional**					
21	**No soy escuchada por los miembros del equipo multidisciplinar**[R]					
22	**Soy reconocida por mi contribución al cuidado de las mujeres por el colectivo médico**					

La escala PEMS-e consta de 17 ítems y dos dimensiones-subescalas: “Apoyo organizacional” (ítems nº 1, 5, 6, 7, 8, 9,10 y 19) y “Capacidades propias y trabajo en equipo” (ítems nº 12, 13, 14, 15, 16, 17, 20, 21 y 22).

Las preguntas de la escala Likert puntúan de 1 a 5 puntos (1 Totalmente de acuerdo, 2 De acuerdo, 3 Ni de acuerdo ni en desacuerdo, 4 en desacuerdo, 5 totalmente en desacuerdo).Las preguntas número 6,8,10,14,19 y 21 puntúan reverso. Se suman las puntuaciones de cada ítem que componen la dimensión y se divide el resultado entre el número de ítems, por lo que el rango de puntuación para cada dimensión es de uno a cinco. A mayor puntuación, mayor percepción del nivel de empoderamiento.

Table S5. Final Spanish Version of *Perceptions of Empowerment in Midwifery Scale (PEMS-e)*
